# The Application of Mesenchymal Stem Cell Therapy in Treating Pulmonary Fibrosis: A Scoping Review

**DOI:** 10.7759/cureus.74611

**Published:** 2024-11-27

**Authors:** Elena Silverstein, Michael Richmann, Delaney Tyl, Ashley Fiaoni, Kylie Pfeifer, Hadi Moussa, Alysia Treacy, Mathew Vigliotta, Michael Schepps, Reena Sheth, Patrick Barry

**Affiliations:** 1 Foundational Sciences, Nova Southeastern University Dr. Kiran C. Patel College of Osteopathic Medicine, Fort Lauderdale, USA; 2 Osteopathic Medicine, Nova Southeastern University Dr. Kiran C. Patel College of Osteopathic Medicine, Fort Lauderdale, USA

**Keywords:** ards (acute respiratory distress syndrome), autologus stem cell transplant, covid 19, lung disease, mesenchymal stem cells, pulmonary fibrosis

## Abstract

Pulmonary fibrosis (PF) is a medical condition that affects the lungs and causes scarring due to the deposition of excess fibrotic tissue. This is often preceded by various causes and can lead to long-term health consequences. The treatment of PF using mesenchymal stem cells (MSCs) to correct lung damage and decrease inflammation is a current focus of research. MSCs are beneficial in inhibiting the immune response and inducing more efficient repair processes, therefore having the potential to be useful in various settings. This review aims to identify the current utilization of MSCs in treating PF in adults. A systematic search was conducted according to the Joanna Briggs Institute Reviewers Manual using Ovid Medline, Embase, and Web of Science to identify studies. Following PRISMA guidelines, eligible peer-reviewed studies that used MSCs to treat adults with PF were identified. The initial search produced 1,836 articles after removing duplicates. Twenty-nine articles met the inclusion criteria. A final analysis of the articles further narrowed the number to eight articles that met all criteria and were relevant to the scoping review's objective. Four studies utilized bone marrow-derived MSCs, two utilized umbilical-derived MSCs, one utilized placenta-derived MSCs, and one utilized adipose-derived MSCs. Of these studies, five administered treatments via an intravenous infusion, two used an endobronchial infusion, and the last utilized an intratracheal approach. The use of MSCs in the treatment of PF in adults was found to be safe with the most common adverse effect reported being fever and chills which resolved a few hours after administration. Although the research regarding MSC use in the treatment of idiopathic PF is relatively new, our results summarize the current sources, route of administration, and current adverse effects. We have shown that future studies with larger sample sizes should be performed to determine long-term outcomes and overall efficacy before clinical practice guidelines become implemented.

## Introduction and background

Mesenchymal stem cells (MSCs) were first studied in human subjects for pharmaceutical use by Hillard Lazarus in 1995 [1]. Because of their anti-inflammatory properties, MSCs were conditionally approved in 2012 for the treatment of graft versus host disease in Canada, New Zealand, and Japan [1]. The use of MSCs to treat chronic degenerative disorders and inflammatory disease has since been justified by these pioneering studies [1]. In the last 10 years, treatment with MSCs has gained in popularity as an option for pathologies such as spinal cord injuries, chronic immune disorders, pulmonary fibrosis (PF), and many other chronic and acute diseases [2]. 

MSCs are multipotent stem cells found primarily in bone marrow, but they can also be found in skin, adipose, umbilical cords, placentas, amniotic fluid, and lung tissue [3,4]. These cells are unique because they have the capacity to differentiate into many cell lines, modulate inflammation, signal immune responses, and initiate tissue repair. In the lungs, MSCs play a crucial role in pulmonary homeostasis and repair [4]. Lung disease, such as PF, interferes with normal function and inhibits the function of lung-resident MSCs [5]. 

PF can be further defined as a medical condition in which there is scarring of the lungs due to the deposition of excess fibrotic tissue [5]. In healthy patients, lung tissue expands and contracts with inhalation and exhalation allowing the blood to become oxygenated while CO_2_ is removed [6]. However, when scar tissue accumulates in the lungs, gas exchange is impaired leaving patients hypoxic with symptoms such as shortness of breath [6]. Milder forms of PF present with symptoms of shortness of breath and cough only after exertion. The most common form of PF is idiopathic, and it is characterized as a chronic, progressive, irreversible, and usually lethal lung disease [7]. Serious respiratory illnesses like severe acute respiratory syndrome coronavirus 2 (SARS‑CoV‑2) can also cause widespread fibrosis due to inflammation and have the potential to be rapidly progressive and fatal [6]. In most cases, the scarring will appear on a radiograph or computed tomography (CT) scan and can be further appreciated with pulmonary function testing [8]. 

The current conservative treatment recommendations for patients diagnosed with PF include both pharmacologic and non-pharmacologic options. The current pharmacologic approach with corticosteroids, colchicine, and cyclophosphamide is widely acknowledged as ineffective by most physicians [9]. Other medications such as pirfenidone or nintedanib have shown some promise and aim to decrease inflammation and slow the progression of the disease [8]. Non-pharmacologic options include oxygen supplementation and pulmonary rehabilitation. The surgical option for patients with progressive disease that is refractory to treatment is ultimately lung transplantation [10]. 

Because of the increase in PF and the lack of adequate treatment, providers and researchers are rapidly studying the efficacy of MSCs in PF and their effect on lung tissue. Many research studies have highlighted the promising efficacy of MSCs as a treatment option for PF patients, however, given this is a new treatment option, few have defined their current clinical use, preferred medium, dosage, and safety profile. This scoping review aims to determine the current clinical application of MSCs in treating adult PF patients so that providers may consider implementing this treatment option into their practice. 

## Review

Materials and methods

Research Question

What are the sources and current uses of MSC therapy in treating adult patients with PF? The purpose of this scoping review was to understand the extent and type of evidence concerning the utilization of MSCs in the treatment of adult patients with PF.

Search Strategy

The electronic databases utilized included Embase, Ovid Medline, and Web of Science. The key search terms incorporated were “mesenchymal stem cell transplantation”, “mesenchymal stem cell”, “mesenchymal progenitor cell”, “multipotent mesenchymal”, “lung disease”, “lung fibrosis”, “lung inflammation”, and “pulmonary fibrosis”. The Boolean operators that we utilized included “and” as well as “or”. Table 1 depicts the search strategy implemented for each database. The search produced 2,435 articles across all three databases (EMBASE, n=756; Ovid MEDLINE, n=277; Web of Science, n=1402). Duplicates were then removed (n=599) leaving 1,836 individual articles to be screened. The search criteria are illustrated in Table 1.

**Table 1:** Article Search Criteria

Search Criteria	
1	('mesenchymal stem cell' or 'mesenchymal stem cell transplantation' or 'mesenchymal stromal cell therapy').mp.
2	('mesenchymal stem cell*' or 'mesenchymal stroma* cell*' or 'mesenchymal progenitor cell*').mp
3	1 or 2
4	'lung fibrosis'.mp.
5	('lung parenchymal fibrosis' or 'lung sclerosis' or 'pneumosclerosis' or 'pulmonary fibrosis' or 'pulmonary sclerosis' or 'lung fibrosis').mp.
6	4 or 5
7	3 and 6
8	limit 7 to humans
9	limit 8 to yr="2008 - 2023"
10	limit 9 to English language

Study Selection

To meet the inclusion criteria, articles had to be peer-reviewed articles, written in English, and taken place between 2008 and 2023. Other criteria included: adult human subjects with PF, worldwide studies, and the application of MSC therapy. Both experimental and quasi-experimental study designs including randomized controlled trials, non-randomized controlled trials, before and after studies, and interrupted time-series studies were included. In addition, analytical observational studies including prospective and retrospective cohort studies, case-control studies, and analytical cross-sectional studies were considered for inclusion. This review also considered descriptive observational study designs including case series, individual case reports, and descriptive cross-sectional studies for inclusion. 

Books, systematic reviews, and scoping reviews were excluded. Other exclusion criteria included: animal studies, subjects who were minors, embryonic stem cells, hematopoietic stem cells, and in vitro studies. 

**Figure 1:**
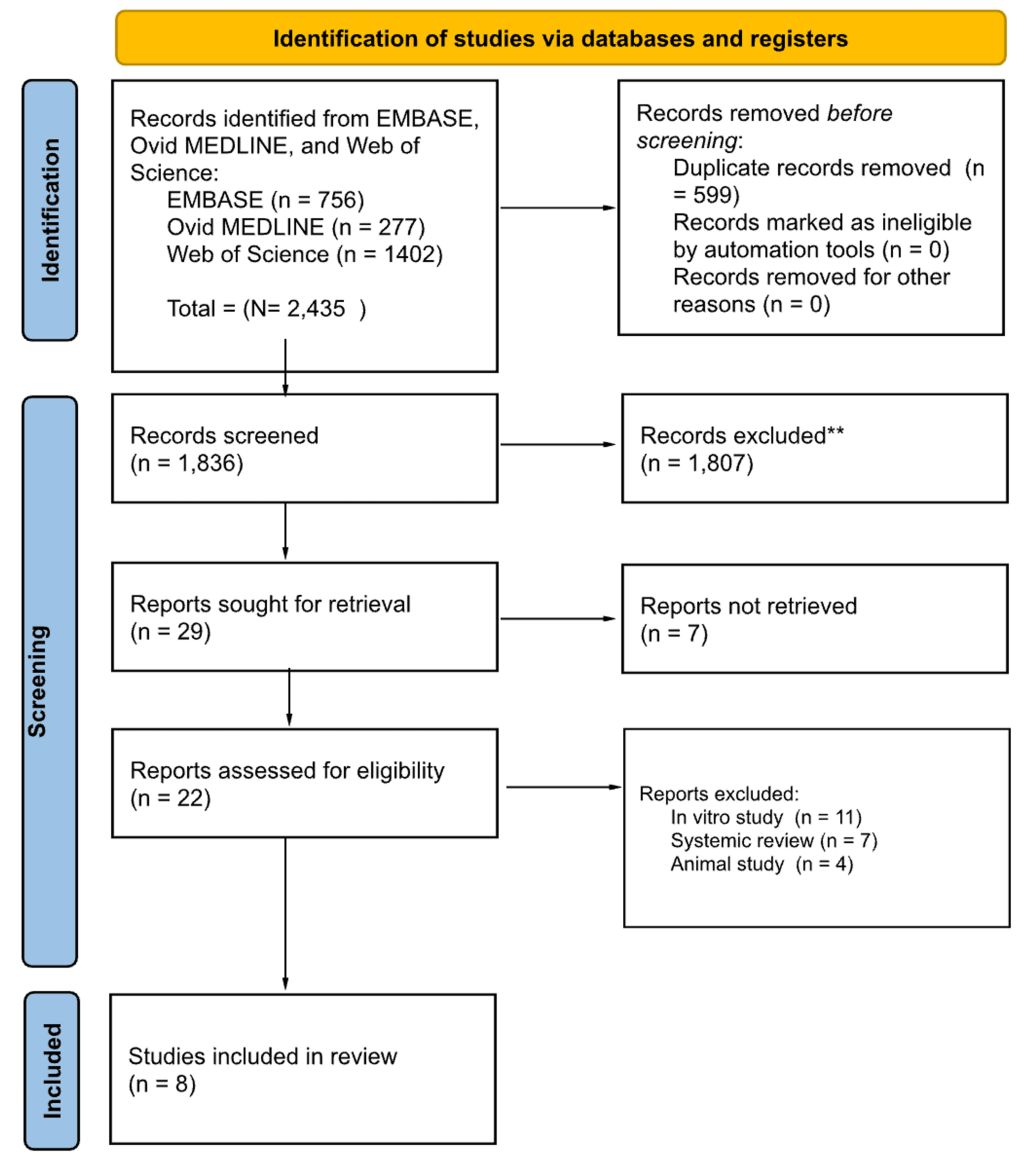
PRISMA Diagram PRISMA: Preferred Reporting Items for Systematic reviews and Meta-Analyses **All records excluded were screened manually without using automatic tools

Using Raayan, nine reviewers in groups of two initially evaluated the titles and abstracts of all the articles, splitting the total number of articles equally among the pairs. Two reviewers then evaluated the full text of all remaining articles and decided which to include for the final review based on our inclusion and exclusion criteria. Any disagreements on study selection were resolved with a designated tiebreaker who made the final decision on whether to include or exclude any articles that the two reviewers disagreed on. We organized our search findings in Figure 1 [11].

Charting the Data

Two reviewers collaboratively created a data-charting form to identify the variables for extraction. Extracted information included characteristics such as country of origin and study type. Additionally, we collected data on population, sample size, methods used, type of MSCs, medium used, and length of treatment. We also documented adverse events, the results of each study, and the limitations of each study. After charting the data, both reviewers engaged in discussions to review the results and updated the data-charting form throughout the process. 

Critical Appraisal

The Joanna Briggs Institute Appraisal Tools (JBI, 2015) were used to evaluate the methodological quality and risk of bias of the included studies. The tools allowed us to classify the articles as high risk of bias (scores less than 50%), moderate risk of bias (scores between 50% and 70%), and low risk of bias (scores above 70%). The level of evidence was classified according to the type of study. Two researchers then read all semifinalist articles in-depth and blindly appraised the articles using the applicable JBI appraisal tools. This appraisal process was then followed by deliberation between two researchers who compared their appraisal scores. A third researcher was brought in to settle any disagreement in appraisal between the two previous reviewers. The relevancy and quality of each article were discussed in detail between the researchers which led to a final consensus on which articles would be included in the article.

Synthesis of Results

We categorized the studies based on the results examined and presented a summary in a table, outlining the settings, populations, and study designs associated with each treatment group. Additionally, we included information on the measures employed and provided a concise overview of the general findings.

Results

Article Selection Process 

Utilizing the predetermined exclusion criteria (animal studies, minors, embryonic stem cells, hematopoietic stem cells, and non-English), nine authors split into three groups and performed the tier 1 abstract review process of the 1,836 articles. The input from a tiebreaker (the tenth author) was used as the final vote in determining the inclusion or exclusion of a study in cases where two reviewers could not agree. The consensus regarding articles relevant for further consideration resulted in the inclusion of 29 articles. 

Tier 2 review was then performed by two reviewers and a tiebreaker. Reviewers read each full-text article (n = 29) and thoroughly screened the remaining articles based on the same inclusion and exclusion criteria. This screening process excluded another 22 articles resulting in eight final articles to be included in our scoping review. Seven articles were excluded due to inaccessibility of their full-text versions. The remaining articles were ultimately excluded because they were either animal studies, in vitro studies, or systematic reviews. The screening and selection process was depicted using a Preferred Reporting Items for Systematic reviews and Meta-Analyses (PRISMA) flowchart. 

Characteristics of the Final Studies

All the final articles in the scoping review originated from various countries (United States, China, Spain, Russia, Korea, Australia, the United Kingdom, and Italy). Study sample sizes ranged from n=1 to n=20. Within study populations, patient backgrounds varied greatly. Various ages were represented both within and among studies, ranging from 33 to 90 years old.

Study Concepts

In seven of the eight studies included in the review, patients had received prior treatment for interstitial PF. One study did not explicitly state any inclusion or exclusion criteria for the study. Two of the eight studies were case reports following a single patient. Other study designs included a single-center, nonrandomized, non-placebo-controlled phase 1 study, a phase 1 clinical trial, a randomized, open-label, placebo-controlled study, an open-label, single-center, non-randomized, dose-escalation phase 1b trial, a non-randomized, no placebo-controlled, unicentric phase 1b clinical trial, and a single-center, non-placebo-controlled, non-randomized phase 1 study.

Safety and Efficacy of MSCs on Humans

Treatment with a single intravenous infusion of allogeneic human cells was determined to be safe in patients with mild to moderate IPF [12,13]. Endobronchial-administered stem cells were also found to be safe in the treatment of IPF [14]. Forced vital capacity (FVC) was reportedly decreased in multiple studies [15,16] (specific percent decline unspecified and 8.1% at 3 months, respectively), while it was increased by 4.7% in the treatment group of another study [17]. Diffusion lung capacity testing (DLCO) was reportedly decreased with the administration of stem cell injections compared to placebo [17,18].

Adverse Effects Associated With MSC Use

No serious adverse effects were mentioned in the majority of the studies [12,14,15,18,19]. Any adverse effects from the studies ranged widely from very severe [16] including hospitalization and death, to less severe [13,17] with patients experiencing fever/chills, increased heart rate, systemic arterial pressure, and bronchitis, respectively).

A summary of the eight articles included in this review is displayed alphabetically by author in Table 2. 

**Table 2:** Summary of the Articles Included in the Review CT: Computed tomography; DLCO: Diffusing capacity for carbon monoxide; FEC: Forced expiratory volume; FVC: Forced vital capacity; MSC: Mesenchymal stem cells

Authors	Purpose	Study Design	Study Population	Methods	Limitations	Key Findings
Averyanov, (2019) [17]	To investigate the safety of infused bone marrow-derived mesenchymal stem cells in patients with idiopathic pulmonary fibrosis	Randomized, open-label, placebo-controlled study	Eleven males and nine females between the ages of 33-74 years with a lung function decline greater than or equal to 10% within the past year.	Patients were split into two random groups of 10 individuals. The first group was given four sets of intravenous MSC infusions (two injections per set) with 12 weeks between sets (infusions were 200 million cells in 400 ml saline. Total MSC = 1.6*10^9). The second group was given a placebo (400ml saline) on the same timeline.	4 out of 20 participants died during the study due to preexisting disease progression	The six-minute walk test increased by 15.3% in the treatment group compared to 2.7% in the placebo group. DLCO change was -1.2% in the treatment group compared to -6.5% in the placebo group. FVC was +4.7% in the treatment group compared to -7.6% in the placebo group.
Campo, (2021) [16]	To establish the safety of endobronchial administration of bone marrow autologous mesenchymal stem cells in patients with idiopathic pulmonary fibrosis	Phase 1 Clinical trial	Thirteen patients without current pregnancy or lactation, emphysema, obstructive or restrictive lung disease (outside of idiopathic pulmonary fibrosis)	Trials were completed in two phases. Phase one was three groups with different doses (10*10^6, 50*10^6, and 100*10^6). Phase two included giving nine patients the highest possible tolerated dose. Following dosing, patients performed pulmonary function testing at 1, 2, 3, 6, and 12 months. CT was performed at 3, 6, and 12 months post-dosing.	Three patients died during the 12-month follow-up period due to disease progression of pulmonary fibrosis	Mean forced vital capacity declined 8.1% at three months. No functional progression of disease at three months was reported by 46% of patients and no progression of disease at 12 months by 23% of subjects.
Chambers, (2014) [13]	To determine the viability and safety of administering placenta-derived human leukocyte antigen-unmatched MSC through an infusion as a treatment method for idiopathic pulmonary fibrosis	Open-label, single-center, non-randomized, dose-escalation phase 1b trial	11 male participants between 40 and 80 years and 4 female participants aged 63.5 (57-75)	In Group 1, four patients received 1×10^6 placenta-derived MSC/kg and in Group 2, four other patients received 2×10^6 MSC/kg if an interim analysis showed satisfactory results. The main objective was safety, and the secondary objectives included evaluating changes in lung function, 6-minute walk distance, gas exchange, and lung fibrosis using various measurements at different time points post-MSC infusion.	Did not assess efficacy because it would be unlikely a single dose of MSCs would be able to instigate a therapeutic effect since MSCs are transiently retained in the pulmonary vascular bed	Ultimately, intravenous MSC therapy is feasible and exhibits a short-term satisfactory safety profile in those with idiopathic pulmonary fibrosis. Findings provide compelling proof of the MSC therapy method's short-term safety in cases of at least moderate fibrotic lung conditions with only minor, self-limiting adverse effects. The results suggest the MSC product in the pulmonary vascular bed does not result in significant adverse clinical consequences.
Chang, (2014) [19]	To study the use of mesenchymal stem cells in the treatment of a case of acute respiratory distress syndrome and pulmonary fibrosis	Case Report	A 59-year-old male with a past medical history of pulmonary tuberculosis, progression from acute respiratory distress syndrome to pulmonary fibrosis	Mesenchymal stem cells were tested for protein markers using flow cytometry. Stem cells were suspended in normal saline (Density = 7.5*10^6/1.5ml). Stem cells were administered through the trachea (Target dose = 1*10^6 kg) into the bronchi evenly (5.5 *10^7 stem cells in each).	The patient died while the study was ongoing	Patient's lung compliance improved from 22.7 ml/cmH20 before the procedure to 26.5, 27.3, and 27.9 ml/cmH20 after 24, 48, and 72 hours following the stem cell administration. Patient later died from hospital acquired infection.
Fishman, (2019) [18]	To assess the correlation between CT-based changes and lung function, specifically DLCO, in people with idiopathic pulmonary fibrosis who were administered different intravenous bone marrow derived MSCs injection dosages	Single-center, non-placebo-controlled, non-randomized phase I study	Six patients were involved in the study and equally split into 2 different groups - 3 subjects for Cohort 1 and 3 subjects for Cohort 2.	Both cohorts studied the effects of Allogeneic Human Cells in subjects with idiopathic pulmonary fibrosis (AETHER) by monitoring at baseline, 24 weeks, and 48 weeks after their first MSC injection. High resolution CT imaging and clinical testing were recorded to compare findings. Cohort 1 got the 2x10^7 hMSC infusion and Cohort 2 received 1x10^8 hMSCs.	The limitations of the study included missing a placebo group, the small sample size, and the non-randomized design.	Cohort 1 subjects who received the 1x10^8 stem cell injection had slower progression in quantitative lung fibrosis and a smaller decrease in DLCO than Cohort 2 who received 2x10^7 hMSC.
Glassberg, (2016) [12]	To evaluate the safety of a single infusion of bone marrow–derived mesenchymal stem cells in patients with IPF	Single-center, nonrandomized, non–placebo-controlled phase I study	Patients between 40 and 90 years with a confirmed idiopathic pulmonary fibrosis diagnosis in line with the guidelines set forth by the American Thoracic Society.	Patients were divided into 3 groups with each group including 3 subjects. Each group was assigned a different intravenous infusion dosage of human bone marrow derived mesenchymal stem cells (20, 100, 200 x 10^6). The study concluded when severe adverse effects were noted. Results were obtained for 60 weeks, followed by another 28 days. Disease progression was recorded as well.	The mesenchymal stem cells were only derived from young men’s bone marrow.	Concludes the safeness of a single intravenous infusion of allogeneic human cells in mild to moderate idiopathic pulmonary fibrosis patients
Tzouvelekis, (2013) [14]	To inspect the safeness of adipose-derived stromal cells - stromal vascular fraction in subjects with mild to moderate idiopathic pulmonary fibrosis	Non-randomized, no placebo-controlled, unicentric phase 1b clinical trial	Twenty patients were screened and upon reviewing the exclusion criteria, 5 were not included due to DLCO<35% and 1 patient refused signing the consent form.	Each patient was administered endobronchial a total of 1.5x10^6 adipose-derived stem cell-stromal vascular fraction F/kg between three doses (0.5x10^6 adipose-derived stromal cells - stromal vascular fraction F/kg per infusion) which were applied monthly. The primary endpoint was to examine the occurrence of treatment-related adverse events during the first year as well as to assess secondary endpoints, including function, exercise capacity, and quality of life measurements at different time intervals.	The study did not examine efficacy or the stem cells' mechanism of action. Also, the researchers noted that the significant improvement of quality of life observed in almost all subjects could be a placebo-effect. Additionally, heterogeneous adipose-derived stromal cells - stromal vascular fraction presents notable methodological challenges because the study design does not allow us to delineate the exact contribution of each of them.	The study achieved its goal in confirming the acceptable safety profile of endobronchial administered autologous adipose-derived stromal cells - stromal vascular fraction. The results contribute to growing scientific insights and set a path for potential future trials focused on effectiveness.
Zhang, (2017) [15]	To assess the potential benefits of human umbilical cord-derived mesenchymal stem cell IV infusion in managing idiopathic pulmonary fibrosis	Case Report	A 56-year-old male with idiopathic pulmonary fibrosis on long term oxygen therapy	Human umbilical cord-derived mesenchymal stem cells (Density = 5 x 10^6 - 1 x 10^7) were infused IV with 20 ml saline. Antibiotics were given via infusion and the subject was monitored after for any severe adverse effects. Results were collected at 6 and 12 months post infusion.	The sample size was small (1 subject)	Forced expiratory volume and FVC values increased after administering infusion. At 12 months, FVC was decreased. The FEV/FVC ratio was increased from 62.3% to 70.5% at six months and 79.9% at 12 months. DLCO improved from 46.3% to 63.1% at 6 months and 78.6% at 12 months.

Discussion

Relevance to Review Question and Objectives

We conducted a scoping review to assess the proposed review question: What are the sources and current use of MSC therapy in treating adult patients with pulmonary fibrosis? We compiled the current data surrounding this novel treatment. Out of the research that met our exclusion and inclusion criteria, it was found that most studies use bone marrow-derived MSCs [12,16-18]. All the studies focused on the use of MSCs in the treatment of idiopathic pulmonary fibrosis (IPF), while two studies focused more specifically on the use of MSCs in the treatment of mild-moderate IPF [13,15]. Nearly all studies administered the treatment intravenously [12,13,15,17-19] in a one-time dose [12,13,15,16,18,19].

Sources of MSCs

Of the four studies that used bone marrow aspirate, one study collected the MSCs from the posterior iliac spine of young healthy men [12], one was an autologous extraction from an unspecified site within the pelvic bones [16], and the remaining two did not specify where the MSCs were gathered from [17,18]. Among the studies, it was found that patients who received autologous samples reportedly had fewer adverse effects and did not have a significant risk of immunologic response [16]. Most importantly, each bone marrow aspirate study concluded the use of bone marrow-derived MSCs is safe in the treatment of IPF. 

Additionally, two studies used human umbilical cord-derived MSCs donated from healthy mothers who had undergone a routine scheduled cesarean section [15,19], while another study used placental-derived MSCs from females who had donated their placenta following an elective cesarean section [13]. Similar to the bone marrow studies, the use of placenta and umbilical cord-derived MSCs was deemed safe in treating IPF. The last study used autologous adipose-derived stromal cells (ADSCs) [14]. The researchers speculated that the use of ADSCs through a lipoaspirate was safer for the patients due to a shortened recovery time and less trauma inflicted on the body during the retrieval phase. Furthermore, the patient received an autologous infusion, minimizing the potential immune response. 

Administration of MSCs

Each study used a variety of MSC concentrations. Studies that compared two or more varying concentrations all concluded that although the cohorts that received the higher doses had more infusion reactions such as fever and chills, they also had a greater opportunity for improvement [12,16,18]. The source of the MSCs also impacted the amount of cells that were able to be infused into the patients. Those from bone marrow were able to administer concentrations ranging from 10 million MSCs to 1.6 billion [12,16-18], umbilical cord-derived MSCs were administered in concentrations from 5 to 55 million MSCs [15,19], placenta-derived MSCs administered 1 to 2 million MSC/kg body weight [13], and finally, ADSCs were administered in 1.5 million ADSC/kg body weight [14]. 

Of the different treatment groups, it was found that those who had received a higher concentration of MSCs experienced more adverse effects, although they were not severe [13,17]. Most adverse effects were related to the infusion time and were reported to be fever and chills [17] or bronchitis [13]. 

When comparing the method of administration, five studies administered the infusions intravenously [12-15,17]. Of those studies, only two reported infusion-related mild side effects including fever and chills [17] and mild systemic adverse effects to the mean arterial pressure, heart rate, and oxygen saturation, all of which resolved within four hours of administration [13]. Two studies administered the treatment through endobronchial infusions. One showed to have severe adverse effects resulting in four hospitalizations and death [16] and one reported no severe adverse reaction [14]. In the former study, the three deaths were deemed due to the natural progression of the disease, complications of hospitalization leading to Legionella pneumonia, and one death of an unknown cause. Due to a lack of statistical significance, the study assumed these events were not due to the infusion itself. The last study used an intratracheal approach with no adverse effects reported [19]. Most importantly, all eight studies concluded MSCs to be a safe modality of treatment for patients suffering from IPF. 

Improvement of Disease

Of those treated with MSCs, those who had more severe PF suffered more severe adverse effects due to treatment [16]. Furthermore, those with an advanced diagnosis of IPF went through unsuccessful MSC treatment which resulted in death [12,16,17,19]. Each of those deaths had been attributed to other causes rather than due to the infusion itself. Those who perished in the study had unforeseen secondary infections such as Legionella pneumonia and empyema leading to septic shock [16,19] as well as a natural progression of the disease [12,17]. It was speculated that had the patients been treated sooner or with more infusions at a higher concentration, the disease progression could have been slowed [19]. 

Implications for Future Research

Given this emerging treatment modality had sparse research, future studies should be done to better understand the best approach to use MSCs in the treatment of IPF. It is important to determine the source of MSCs that produces the least adverse effects but also provides the greatest outcome to patients. Interestingly, only one study used autografts [14]. While this study reported there to be fewer adverse events due to a lack of an immunologic response, it would be beneficial to run additional research to establish the overall efficacy of autograph MSCs compared to allograft sources. Additionally, the mode of administration should be further researched to find a balance between patient safety and efficacy. The majority of the studies used intravenous routes for treatment [12,13,15,17-19], and few chose endobronchial or endotracheal modes [14,16,19]; however, we propose a comparative study to be done to compare the routes of administration. Finally, the current studies showed there might be benefits to higher dosing of MSCs, however, there were greater risks of adverse reactions [12,13,17]. Given the sparse research regarding the most appropriate dosing, it is important to further analyze the risk-benefit relationship between dosing and adverse reactions. 

Limitations

There were several limitations when conducting the scoping review. While Critical Appraisal was used to reduce the amount of bias in the articles we used and determine the relevancy and quality of each article, this process was not perfect. Although three individual researchers were involved in each process, it is possible a different set of researchers in our team doing the same Critical Appraisal would have resulted in a different outcome. This could have led to different research articles being approved for our final review, subsequently changing our results. Another limitation was the accuracy of our Tier 1 review; 1,836 articles were screened manually by 10 separate researchers. This could introduce room for bias against certain articles that would not have been accounted for because most of these articles would not have reached the Critical Appraisal stage of our review. 

For the time being this scoping review should not be used for clinical guidelines or as a recommendation for treatment since each research article’s sample sizes were 20 participants [14,17] or less [12,13,15,16,18,19]. Furthermore, the research in these articles was all conducted recently, being performed less than 10 years ago; this would indicate that this specific use of MSCs to treat IPF was still a newer method and requires more experiments with much larger sample sizes to provide more reliable and accurate results.

## Conclusions

While the research regarding MSC use in the treatment of idiopathic PF is relatively new, it presents an exciting prospective form of treatment. Most studies used bone marrow-derived MSCs; however, there were other sources used including umbilical-derived MSCs, placental-derived MSCs, and adipose-derived MSCs. Each source of MSCs came with limitations including the amount of MSCs obtained for clinical use and potential adverse reactions. Finally, the route of administration was mostly through intravenous methods, but it was shown that endobronchial and endotracheal administration could be performed. With further research, the most effective source of MSCs can be determined, the best dosage to yield the greatest results could be established, and the best method of administration could be solidified. 
