# Correction: The Application of Mesenchymal Stem Cell Therapy in Treating Pulmonary Fibrosis: A Scoping Review

**DOI:** 10.7759/cureus.c338

**Published:** 2025-10-01

**Authors:** Elena Silverstein, Michael Richmann, Delaney Tyl, Ashley Fiaoni, Kylie Pfeifer, Hadi Moussa, Alysia Treacy, Mathew Vigliotta, Michael Schepps, Reena Sheth, Patrick Barry

**Affiliations:** 1 Foundational Sciences, Nova Southeastern University Dr. Kiran C. Patel College of Osteopathic Medicine, Fort Lauderdale, USA; 2 Osteopathic Medicine, Nova Southeastern University Dr. Kiran C. Patel College of Osteopathic Medicine, Fort Lauderdale, USA

This article has been corrected to fix the following typo in the PRISMA diagram: 'Reports excluded' corrected from 22 to 14.

